# Targeting regulation of VEGF by BPTF in non-small cell lung cancer and its potential clinical significance

**DOI:** 10.1186/s40001-022-00935-1

**Published:** 2022-12-19

**Authors:** Meng Dai, Chunyu Hua, Mingqin Wang, Li Gao, Ling Jiang, Yuan Liu

**Affiliations:** 1 Dalian Municipal Central Hospital, Dalian University of Technology, Dalian, 116011 China; 2 China Medical University Graduate School, Shenyang, China; 3 Institute of Cancer Stem Cell, Dalian Medical University, Dalian, China

**Keywords:** BPTF, VEGF, Angiogenesis, NSCLC, Bevacizumab, Biomarkers

## Abstract

**Purpose:**

VEGF facilitates tumor angiogenesis, and bevacizumab targeting VEGF is used in anti-tumor therapy. It is meaningful to clarify the upstream regulatory mechanism of VEGF. BPTF is important in chromosomal remodeling, and promotes the progression of tumors. However, its role in promoting tumor angiogenesis by targeting VEGF has not been fully reported. This study aims to elucidate the expression regulation of VEGF by BPTF and its clinical significance in NSCLC.

**Methods:**

1. BPTF siRNA and shRNA plasmids were used to reduce the expression of BPTF by transfection in vivo and in vitro. BPTF, VEGF and CD144 expressions were examined by immunofluorescence and Western Blot. 2. The expressions of BPTF, VEGF, CD144 and CD31 were detected in lung adenocarcinoma samples by immunofluorescence, Western blot and immunohistochemical staining. 3. 26 lung adenocarcinoma patients treated by bevacizumab were divided into 2 groups according to the treatment efficacy. BPTF and VEGF expressions were analyzed.

**Results:**

1. BPTF knockdown inhibited the expression of VEGF and CD144 in vivo and in vitro. 2. Compared with para-cancer tissues, BPTF, VEGF, CD144 and CD31 were highly expressed in lung adenocarcinoma. 3. In 75 lung adenocarcinoma specimens, BPTF and VEGF overexpression was correlated with lymph node metastasis and clinical stage. The 5-year survival rate of patients with BPTF and VEGF low expression was higher, and BPTF expression was positively correlated with VEGF expression. 4. Among 26 patients treated with bevacizumab, the patients with BPTF overexpression are more sensitive to the treatment.

**Conclusions:**

BPTF positively regulates VEGF expression and its high expression predicts a better efficacy of bevacizumab treatment in NSCLC.

## Introduction

It is well known that neovascularization plays an extremely important role in the growth, metastasis and prognosis of malignant solid tumors, and the structural characteristics of neovascularization make distant metastasis of malignant tumor tissues possible [1]. Quantification of neovascularization in malignant solid tumors is considered to be an important independent prognostic marker [2]. Angiogenesis is a complex process involving a variety of molecules, and VEGF plays a central role in tumor angiogenesis and is the most active and specific angiogenic factor known at present [3]. Accordingly, bevacizumab targeting VEGF has been widely used in the oncology field. Therefore, it is of great significance to further clarify the upstream regulatory mechanism of VEGF.

However, the regulatory mechanism of VEGF expression has not been fully clarified. Current studies have found that HIF-1 is the main transcription factor regulating VEGF, and activates this gene to initiate the transcription of VEGF in the hypoxia environment. In addition, VEGF expression is also regulated by the expression products of some proto-oncogenes and tumor suppressor genes, such as C-SRC. In eukaryotes, DNA does not exist naked, but forms nucleosomes by wrapping histone cores. The presence of this form prevents DNA from interacting with transcription factors and basic transcription devices, thus blocking transcription. Therefore, chromatin remodeling is the basic and most important step in gene expression regulation. Through chromosomal remodeling, DNA can combine with trans-acting factors to initiate or inhibit transcription. The hot tumor factor CBP falls into this category. As the largest subunit of nucleosome remodeling factor complex (NURF), BPTF plays a key role in chromosomal remodeling. Back in 2006, two papers serialized in *Nature *detailed its role in normal cells. By binding to methylated and acetylated histone sites, NURF catalyzes ATP-dependent nucleosome slippage and subsequently promotes chromatin transcription [4, 5]. As an important transcription factor, the overexpression of BPTF leads to the transcription of tumor-related genes and ultimately enables the normal cells to have the ability to proliferate and metastasize indefinitely, namely the formation of malignant tumors. Since 2015, the carcinogenic effects and mechanisms of BPTF in melanoma, non-small cell lung cancer, colorectal cancer, hepatocellular carcinoma and childhood glioblastoma have been reported successively [6]. However, there have been no reports on the role of BPTF in promoting tumor angiogenesis by targeting VEGF.

This study aimed to clarify the regulation of VEGF expression by BPTF in non-small cell lung cancer, the correlation between the expression of BPTF and clinicopathological parameters and patient prognosis, and the relationship between the expression of BPTF and the efficacy of bevacizumab treatment. Therefore, all the results could provide a theoretical and experimental basis for further revealing the biological functions and the related molecular mechanisms of BPTF in cancer, especially in lung cancer.

## Patients/materials and methods

### Cell lines and cell culture

Human non-small cell lung cancer A549 and NCI-H460 cells were purchased from ATCC (American Type Culture Collection). A549 and NCI-H460 were cultured in DMEM and RPMI-1640 medium supplemented with 10% FBS, respectively. All cells were placed in an incubator (Thermo Fisher Scientific) at 37 °C containing 5% CO2.

### Immunofluorescence

Clinical tissue sections of patients with non-small cell lung cancer or tumor tissue subcutaneously implanted in nude mice were taken, and the sterilized cover glass was placed in a six-well plate, and the sample was fixed with 4% paraformaldehyde for 20 min after the cell fusion rate reached more than 80%. After washing with PBS for 3 times, discard PBS, then added 1 ml 0.2% Triton-100. Later washed with PBS for 3 times, added 1 ml 10%BSA for 30 min after permeating at room temperature for 5 min. The primary antibody was diluted with PBS and incubated with the sample at 4 °C overnight. After washing with PBS, wash solution was drained with absorbent paper, and then fluorescence secondary antibody coupled with fluorescein isothiocyanate and rhodamine was added and incubated at room temperature for 1 h, away from light. After rinsed with PBS for two times, 5 mg/ mL DAPI was diluted at a ratio of 1:10,000 and then dropped onto the slide to re-stain the nuclear. After 5 min, the slides were washed and sealed, and then the samples were observed and photographed under a Leica confocal microscope.

### Western blot analysis

Lysates containing protease inhibitors were added to the collected cells or tissues. After wholly lysing, the supernatant was collected after centrifuging at a speed of 14000rcf in a low-temperature high-speed centrifuge for 10 min, and 5ul was removed for protein quantification. Firstly, we added sample 20 μg to 10% SDS-PAGE gel, and performed electrophoresis at 70 V constant pressure to bromocresol blue indicator to the junction of concentrated gel and separated gel, then used 90 V constant pressure until bromocresol blue indicator to the bottom of gel. Secondly, we took out the gel, cut the target band according to the protein marker, and cut the polyvinylidene fluoride membrane with the same size as the gel and activated it with methanol. Finally, we organized the components in the sequence of black plate/fiber pad/filter paper/gel-polyvinylidene fluoride membrane/filter paper/fiber pad/black plate, clamped the plate and put it in the transfer tank, filled the transfer liquid and started the transfer. After sealing polyvinylidene fluoride with 8% skim milk at room temperature for 2 h, the membrane were separately combined with specific antibodies BPTF(1:2000, Abcam), VEGF(1:1000, Abcam), VE cadherin(1:1000, Abcam), CD31(1:1000, Abcam), GADPH(1:20,000, Proteintech) and β actin(1:500, Proteintech) and incubated at 4 °C overnight. TBST was rinsed for 3 times and incubated with HRP labeled secondary antibody at room temperature for 2 h. After full washing of TBST, protein bands were detected by enhanced chemiluminescence solution.

### Transient transfection of siRNA

SiRNA was synthesized by Shanghai Genepharma (China). The company designed nonspecific siRNA and three BPTF-siRNAs. Among them, two of the BPTF si-RNAs knocked down BPTF were obvious. Their sequences were: nonspecific siRNA: 5′-UUC UCC GAA CGU GUC ACG UTT-3′ and 5′-ACG UGA CAC GUU CGG AGA ATT-3′; BPTF-siRNA-1 (BPTF-homo-1550): 5′-GGU CCA ACU UGC AGA AUU ATT-3′ and 5′-UAA UUC UGC AAG UUG GAC CTT-3′; BPTF-siRNA-2 (BPTF-homo-6959): 5′-GAC CCA AAC AAC UGU UUC ATT-3′ and 5′-UGA AAC AGU UGU UUG GGU CTT-3′. The transfection process was as follows: first, cells were implanted into each well of a 6-well tissue culture plate, and the cell fusion rate was estimated at 60–80% on the next day. Meanwhile, reagents mixed with RNAiMax (Invitrogen, Carlsbad, USA) and BPTF siRNA were placed in the cells that were still suspended according to the instructions of RNAiMax. The next day, when the cells adhered to the culture plate, we replaced the medium containing 10% FBS but no antibiotics with a new medium supplemented with 10% FBS but containing antibiotics. After 48 or 72 h, cells were collected.

### In vivo tumor model

Animal experiments were carried out in accordance with the National Hygienic Guidelines for the Use of Experimental Animal Care approved by APF Experimental Animal Center of Dalian Medical University. The following study also adhered to the ARRIVE guidelines. Male nude mice 4 to 6 weeks old were used in this study. Their average weight was 20 g. They were fed in SPF animal laboratory where food and water were freely available. A549 cells (5 $\times$ 10^6^) were placed subcutaneously under the left arm of nude mice. About 14 days later, the tumor had grown to 5 mm $\times$ 5 mm (length $\times$ width), and the nude mice were randomly divided into two groups. There were five nude mice in each group. There was no difference in tumor volume and size of nude mice between the two groups. Group 1 was the control shRNA and group 2 was the BPTF shRNA. According to the Lipofectamine 3000 specification, shRNA plasmids were transfected into tumors. 25ug shRNA was injected in 100 μl mixture every 4 days for 28 consecutive days. Used a digital caliper, we calculated the tumor volume by "volume = (width^2^ $\times$ length)/2". Finally, nude mice were killed in the operating room of SPF animal laboratory by cervical dislocation for further detection of related proteins by Western blot.

### Clinical tissue sample experiment

#### Case data

Collected 75 cases of lung adenocarcinoma were directly purchased tissue microarray chips from Shanghai outdo biotech CO., LTD. Normal adjacent lung tissue was used as control. All the patients were patients with lung adenocarcinoma who received surgical treatment from January 2004 to December 2010, and did not receive radiotherapy or chemotherapy before surgery. All the patients had relatively complete clinicopathological data, including age, gender, family history, and postoperative pathological information.

A total of 26 patients receiving bevacizumab in Dalian Central Hospital from January 2018 to January 2021 were collected. Approval was obtained from the ethics committee of Dalian Municipal Central Hospital. The procedures used in this study adhere to the tenets of the Declaration of Helsinki. Informed consent was obtained from the participants.

#### Follow-up

For 75 patients of Xinchao Company, follow-up was conducted through telephone follow-up and out-patient review, and the follow-up contents were mainly survival, postoperative adjuvant treatment, recurrence, imaging examination and tumor marker level. The follow-up period ended 5 years after the onset of the disease, and the final deadline was December 2016. The end point event was defined as death from any cause. Overall survival was from the first postoperative day to the time of death from any cause.

For the follow-up of 26 patients treated with bevacizumab, we followed them up through out-patient and in-patient review, including survival, postoperative adjuvant treatment, recurrence, imaging examination and tumor marker level. Follow-up ended in March 2022.

##### Criteria for efficacy evaluation of bevacizumab

Efficacy evaluation was performed after two cycles (6–8 weeks) of bevacizumab, and efficacy evaluation was performed according to RECIST 1.1 criteria, which were divided into complete response (CR), partial response (PR), stable (SD) or progressive disease (PD). (1) Complete remission (CR): except for nodular disease, all target lesions disappeared completely. All target nodules must be reduced to normal size (short axis < 10 mm). All target lesions should be evaluated. (2) Partial response (PR): the total diameter of all measurable target lesions was ≥ 30% below baseline. The sum of the target nodules used the short diameter, while the sum of all other target lesions used the longest diameter. All target lesions should be evaluated. (3) Disease progression (PD): take the minimum sum of the diameter of all target lesions measured during the whole experimental study as the reference, and increase the diameter and relative value by at least 20% (if the baseline measurement value is the minimum, the baseline value is the reference); in addition, an absolute increase of at least 5 mm in diameter and diameter must be met (the presence of one or more new lesions is also considered disease progression). (4) Stable disease (SD): the decrease degree of target lesions does not reach PR, and the increase degree does not reach PD level, which is between the two. The minimum sum of diameter can be used as a reference in the study. Patients were divided into two groups based on their response to treatment: those with good response (complete response, partial response, or stable disease) and those with poor response (progression).

#### IHC detection methods and evaluation criteria for BPTF and VEGF

Immunohistochemical staining (IHC): After xylene dewaxing, gradient hydration, removal of endogenous Peroxidase activity, high-pressure antigen repair, PBS buffer washing, IHC oil marker circled around the section, and then sheep serum was dropped to seal the section for 30 min. Primary antibody incubation: BPTF (purchased from Abcam) was diluted with 1: 500 and VEGF (purchased from Proteintech) at 1:800. The whole section was covered and incubated overnight at 4℃. Then washed with PBS buffer 4 times, 3 min each. Secondary antibody incubation: added secondary antibody drops and incubated at room temperature for 1 h. Washed with PBS buffer 4 times, 3 min each. DAB was used for 2-3 min, and the reaction was terminated by washing with water. After 1-2 min, the differentiation of alcohol hydrochloride was 1-3 s. Dehydrated and dried, sealed with neutral resin, and observed.

Positive results were assessed by two senior pathologists based on the degree of staining. According to the dyeing depth, the results were divided into four grades."-" is rated as 0 points, " + " as 1 point, " +  + " as 2 points, " +  +  + " as 3 points. According to the staining area of the tissue:25% is rated as 1 point, 25%-50% as 2 points, 50%-75% as 3 points, and over 75% as 4 points. The total score was the staining depth score $\times$ staining area score, and the highest score was 12. We defined high expression with a score above 7, and low expression with a score below 6.

#### Statistical analysis

The correlation between BPTF high expression and N (number of lymph node metastasis) and clinical stage was analyzed according to Pearson Chi-square test. Kaplan–Meier test and log-rank test were used to analyze the difference in overall survival of BPTF and VEGF with both high and low expression. The relationship between BPTF or VEGF expression and bevacizumab efficacy was analyzed by Pearson Chi-square test. The subject involved all statistics, and the test level was 0.05, P < 0.05 was statistically significant.

## Results

### Expression correlation of BPTF, VEGF, VE cadherin (CD144) and CD31 was detected by Western blot and immunofluorescence at cell and nude mice levels

**Fig. 1:**
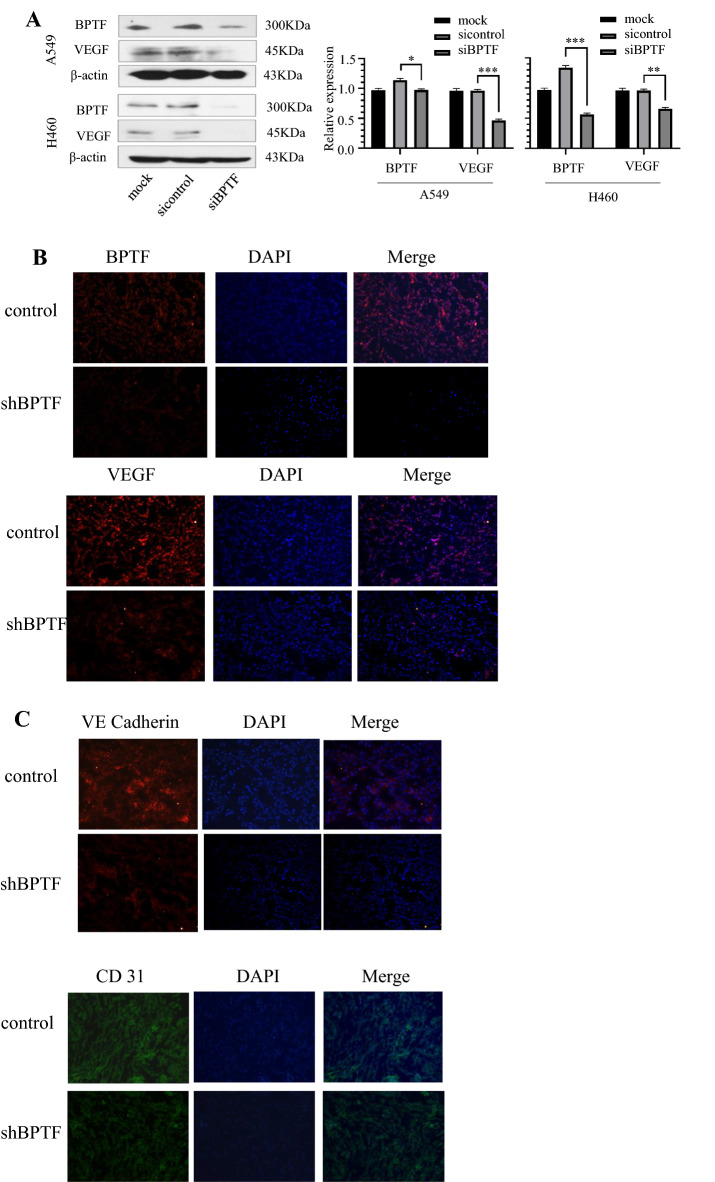
The expression levels of VEGF and VE cadherin related to tumor angiogenesis in NSCLC were detected by knocking down BPTF at both cell and animal levels. **a** BPTF siRNA was transfected into A549 and NCI-H460 cell lines to knock down BPTF, and the expressions of BPTF, VEGF and VE cadherin were detected by Western blot and immunofluorescence. **b** Nude mouse model was established, A549 cell line was planted, BPTF was knocked down by transfection BPTFshRNA, and the nude mice were killed 28 days later, and the expression of BPTF and VEGF was detected by immunofluorescence method. **c** In this nude mouse model, the expression of VE cadherin associated with angiogenesis was further detected after BPTF knockdown

By transfecting BPTF siRNA, BPTF was, respectively, knocked down in A549 and NCI-H460 cells, and VEGF protein expression was also detected to decrease (Fig. 1a). Further, in vivo experiments were conducted in nude mice. A549 cells were implanted subcutaneously in nude mice. After 14 days of tumor growth to 5 × 5 cm, the animals were divided into two groups: one group was injected with control BPTF plasmid and the other group was injected with BPTF shRNA plasmid, once every 4 days for a total of 28 days. Tumors were removed, and the expression of BPTF and VEGF in tumor tissues of mice was detected by immunofluorescence method. The results showed that compared with the control group, the expression level of VEGF decreased in BPTF knockdown group (Fig. 1b). We further detected the expression of VE cadherin and CD31 (Fig. 1c), and found that following with the down-regulated expression of BPTF, the expressions of VE cadherin and CD31 also decreased. The results showed that BPTF knockdown inhibited VEGF, CD31 and VE cadherin associated with tumor angiogenesis in non-small cell lung cancer.

### Expressions of BPTF, VEGF, VE cadherin and CD31 in postoperative cancer tissues and fresh adjacent tissues of patients with first-stage lung adenocarcinoma were detected by immunofluorescence and Western blot

**Fig. 2:**
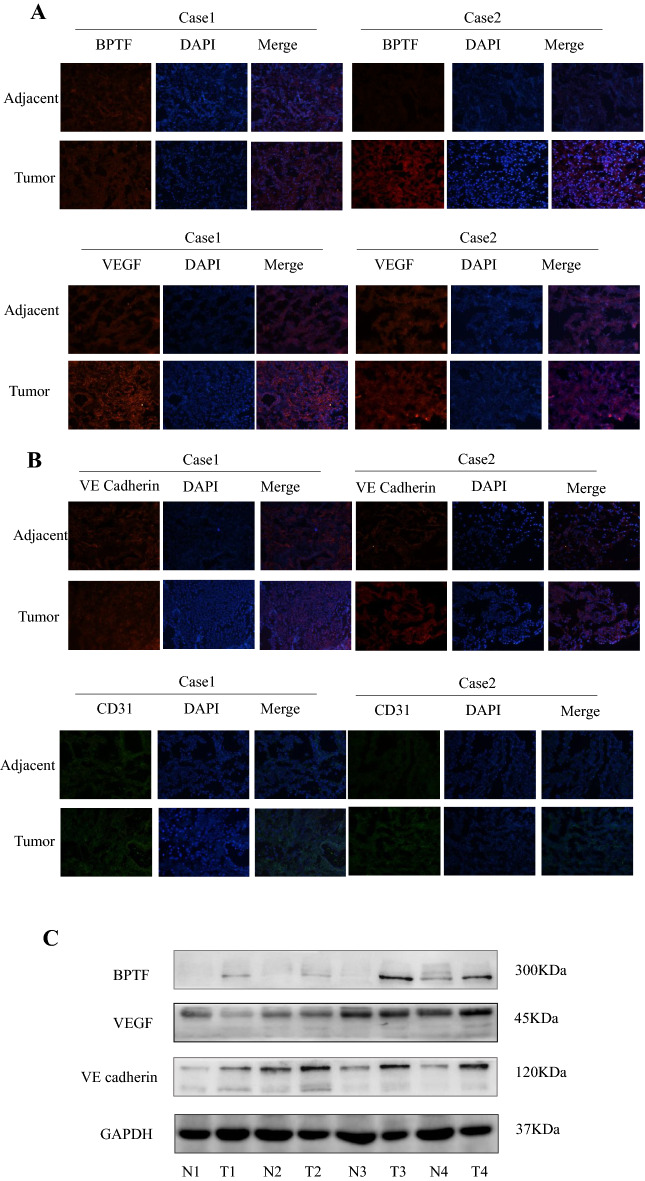
Expressions of BPTF, VEGF, VE cadherin and CD31 were detected in postoperative clinical tissue samples of human lung adenocarcinoma. **a** Fresh postoperative cancer tissue and para-cancer tissue samples of 10 patients with lung adenocarcinoma were randomly selected, and the expression levels of BPTF and VEGF were detected by immunofluorescence method, among which two representative cases were selected. **b** The expression of angiopoiesis-related CD31 and VE cadherin was detected by the same method, and two representative cases were selected. **c** Western blot was used to detect the expressions of BPTF, VEGF and VE cadherin in cancer tissues and adjacent tissues of 10 patients with lung adenocarcinoma. “N” represents relatively normal tissue adjacent to cancer, and “T” represents tumor tissue

Cancer and para-cancer specimens of 10 patients with primary adenocarcinoma after operation were randomly selected as controls. Figure 2a shows the immunofluorescence diagram of BPTF and VEGF, which showed higher expression of BPTF and VEGF in cancer tissues compared with para-cancer tissues. Further analysis of vascular endothelial cadherin (VE cadherin, CD144) and endothelial tissue biomarker CD31 showed that CD144 and CD31 were higher in cancer tissues than in para-cancer tissues (Fig. 2b). Figure 2c shows the expression of BPTF, VEGF and CD144 in cancer and para-cancer tissues collected from 4 patients detected by Western blot. The results still showed that the expression of BPTF, VEGF and CD144 in cancer tissues was higher than that in para-cancer tissues. The results showed that compared with the adjacent tissues, BPTF, VEGF, VE cadherin and CD31 were highly expressed in cancer tissues.

### Expression, clinicopathological characteristics, prognosis and survival analysis of BPTF and VEGF in postoperative lung adenocarcinoma tissue samples, and statistical analysis of their correlation

A total of 75 samples (tumor and adjacent tissue) were collected from the lung adenocarcinoma microarray chip of Shanghai Xinchao Company. The case collection period was 5 years from June 2004 to June 2009. All the patients were from third-class hospitals in Shanghai. They had not received radiotherapy or chemotherapy before surgery, and had complete clinical data. The follow-up ended in September 2014.

**Fig. 3:**
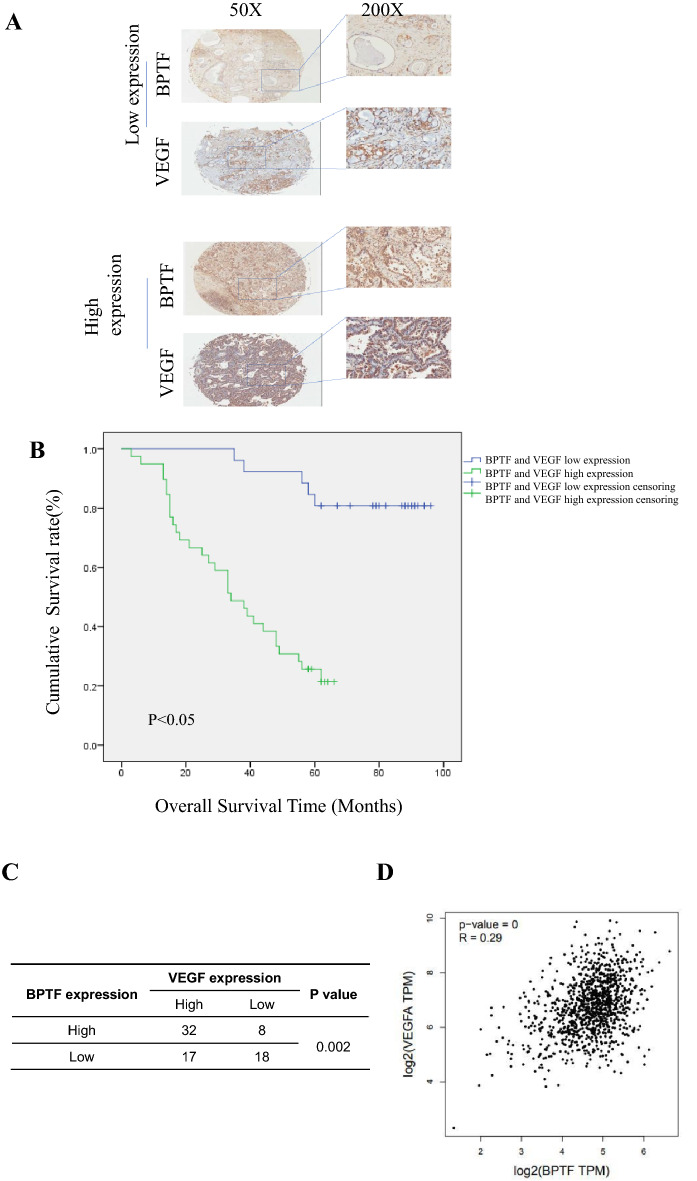
Tissue samples from 75 patients with lung adenocarcinoma after operation were collected, and the expression of BPTF and VEGF was detected by immunohistochemical method. The clinicopathological characteristics, prognosis and survival of BPTF and VEGF were statistically analyzed. **a** Immunohistochemistry was used to detect the expression of BPTF and VEGF in cancer and adjacent tissues. **b** Survival curves of patients with both high and low expression of BPTF and VEGF. As shown in the figure: high expression of both indicated shorter survival time and lower survival rate than low expression of both. **c** Chi-square test was used to analyze the correlation between BPTF and VEGF in this lung adenocarcinoma tissue, and the results showed that *P* < 0.05 was statistically significant, indicating a certain correlation between the two. **d** The correlation between BPTF and VEGF was analyzed in GEPIA database, and the correlation coefficient was 0.29

In order to study the expression of BPTF and VEGF in non-small cell lung cancer, the above lung adenocarcinoma tissue samples were selected and the expression of BPTF and VEGF was detected by immunohistochemical method. The results were evaluated by two deputy chief physicians and pathologists (Fig. 3a).

Table 1 shows the related clinicopathological features. There were 75 pathological tissues of lung adenocarcinoma, aged 35–80 years, with an average age of 59 years, including 45 males and 30 females, 35 patients with lymph node metastasis and 40 patients without lymph node metastasis, 37 patients with TNM stage T1+T2, and 38 patients with T3+T4. There were 17 patients in clinical stage I, 22 in clinical stage II, 19 in clinical stage III, and 17 in clinical stage IV. The positive rate of VEGF in lung adenocarcinoma was 65% and that in para-cancer was 11.8%, the difference between the two groups was statistically significant (*P* < 0. 05); the positive rate of BPTF in lung adenocarcinoma was 52%, and the para-cancer positive rate was 3.2%, the difference was statistically significant (*P* < 0.05). According to Pearson Chi-square test analysis, BPTF overexpression and VEGF overexpression were statistically significant with lymph node metastasis and clinical stage, respectively, while there was no significant correlation with patient age, gender and tumor size.

**Table 1:** VEGF and BPTF expression in tumor tissues from 75 patients with lung adenocarcinoma and the correlation between their expression and the clinicopathological features

Clinicopathological features		Case number	VEGF			BPTF		
			** + **	**−**	*P* value	+	−	*P* value
Age (years)	< 60	31	20	11	0.901	15	16	0.599
	≥ 60	44	29	15		24	20	
Sex	Male	45	30	15	0.766	26	19	0.22
	Female	30	19	11		13	17	
Lymphatic metastasis	Yes	35	28	7	**0.013**	25	10	**0.002**
	No	40	21	19		14	26	
Clinical stages	I	17	11	6	**0.033**	5	12	**0.01**
	II	22	10	12		10	12	
	III	19	17	2		10	9	
	IV	17	11	6		14	3	
T	T1 + T2	37	22	15	0.292	22	15	0.202
	T3 + T4	38	27	11		17	21	

Furthermore, Kaplan–Meier method was used to describe the survival curve, and log-rank test was used for statistical analysis. The test level was *P* < 0.05 was statistically significant. The results showed that the 5-year survival rate was 78% when both of them were low expression in lung adenocarcinoma, while the 5-year survival rate was only 23% when both of them were high expression in lung adenocarcinoma, the difference between the two groups was statistically significant (*P* < 0. 05). This indicated that the high expression of both indicated worse prognosis of patients (Fig. 3b). Further analysis of the correlation between BPTF and VEGF showed that < 0.05 was statistically significant (Fig. 3c), and there was a certain correlation between them, with a correlation coefficient of 0.29 (Fig. 3d).

### The clinical significance of the positive correlation between BPTF and VEGF in evaluating the efficacy of bevacizumab in patients with lung adenocarcinoma

**Fig. 4:**
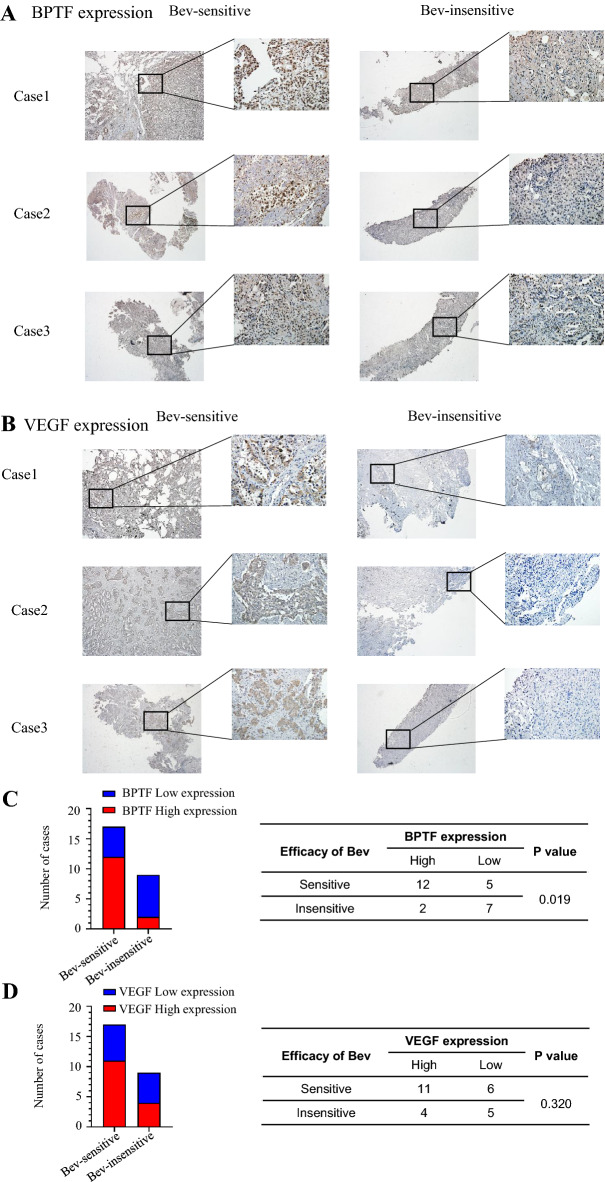
Clinical significance of the positive correlation between BPTF and VEGF in evaluating the efficacy of bevacizumab in patients with lung adenocarcinoma. A total of 26 patients treated with bevacizumab were divided into two groups according to bevacizumab with good efficacy (sensitive) and poor efficacy (non-sensitive). **a** Immunohistochemistry was used to detect the expression level of BPTF between the two groups. Among them, three pairs of representative images were selected with 40X and 200X scales, respectively. **b** Immunohistochemistry was used to detect the expression level of VEGF between the two groups. Among them, three pairs of representative images were selected with 40X and 200X scales, respectively. **c** Expression of BPTF and VEGF in bevacizumab sensitive group and non-sensitive group. As shown in the chart: In the bevacizumab sensitive group, 12 cases had high expression of BPTF, and 5 cases had low expression. In the insensitive group, there were 2 cases with high expression of BPTF and 7 cases with low expression, *P* = 0.019 by Chi-square test, showing statistical difference. **d** VEGF expression was also detected in the two groups. The results showed that in the bevacizumab sensitive group, 11 cases had high VEGF expression and 4 cases had low VEGF expression; in the insensitive group, there were 6 cases of high VEGF expression and 5 cases of low VEGF expression. Although there was a higher VEGF expression trend in the bevacizumab sensitive group, the Chi-square test showed no statistical difference, *P* = 0.32

Bevacizumab is a monoclonal antibody that inhibits angiogenesis, which can inhibit the bioactive form of VEGF [7, 8]. Therefore, we collected 26 clinical tissue samples from patients with lung adenocarcinoma who were still progressing after surgery, chemotherapy and targeted therapy, and treated with bevacizumab, and tested whether the expressions of BPTF and VEGF were correlated with bevacizumab efficacy. The results showed that BPTF was generally highly expressed in the bevacizumab sensitive group, and lowly expressed in the bevacizumab insensitive group (Fig. 4a). Similarly, VEGF expression was relatively higher in the bevacizumab sensitive group than in the non-sensitive group (Fig. 4b). Further, we statistically analyzed the number of cases with high and low expression of BPTF and VEGF in the bevacizumab sensitive and insensitive groups, respectively. The results showed that in the bevacizumab sensitive group, there were 12 cases with high expression of BPTF and 5 cases with low expression. In the insensitive group, there were 2 cases with high expression of BPTF and 7 cases with low expression, *P* = 0.019 by Chi-square test, showing statistical difference (Fig. 4c). Similarly, VEGF expression was statistically analyzed in the bevacizumab sensitive and insensitive groups. The results showed that in the bevacizumab sensitive group, there were 11 cases of high VEGF expression and 4 cases of low VEGF expression. In the insensitive group, there were 6 cases of high VEGF expression and 5 cases of low VEGF expression. Although there was a higher VEGF expression trend in the bevacizumab sensitive group, the Chi-square test showed no statistical difference (*P* = 0.32) (Fig. 4d). The use of bevacizumab is not based on VEGF expression, and the specific mechanism of this phenomenon needs to be further studied.

## Discussion

As the largest subunit of nucleosome remodeling factor complex, BPTF plays a key role in chromosome remodeling. The overexpression of BPTF promotes tumor occurrence and development, and its mechanism has been partially elaborated in melanoma, non-small cell lung cancer, bladder cancer, colorectal cancer and hepatocellular carcinoma. For example, as a downstream target gene of MITF (microphthalmia-associated transcription factor), BPTF mediates the promotion effect of MITF on melanoma [9]. The direct interactions of BPTF with c-MYC recruit c-MYC to histone methylation and acetylation modification sites and activate transcription of downstream target genes [10]. The high expression of BPTF can maintain the stemness of tumor cells and promote the progression of hepatocellular carcinoma by targeting activation of human telomerase reverse transcriptase. However, so far, BPTF has not been reported to promote tumor proliferation and lymphatic metastasis by participating in tumor angiogenesis.

Neovascularization plays an extremely important role in the growth, metastasis and prognosis of malignant solid tumors. The structural characteristics of neovascularization make distant metastasis of malignant tumor tissues possible [1, 11, 12]. VEGF plays a crucial role in angiogenesis via promoting activation of key downstream signaling pathways. It can bind to VEGFR2 receptor on vascular endothelial cells to activate downstream Notch signaling pathway, thereby regulating endothelial cell differentiation and inducing angiogenesis and tubular formation, thus providing nutrition for tumor cells to meet the needs of tumor cell growth and accelerate the development process of tumor [13–16]. In addition, VEGF may directly promote the growth of tumor stem cells. Further studies have shown that VEGFR-3 ligand binds to and activates VEGFR-3, induces, controls and regulates lymphangiogenesis. The way of tumor lymphatic metastasis comes from VEGF-C and VEGF-D activated by tumor cells, which are upregulated by tumor cells. The upregulated VEGF-C and VEGF-D further induce the opening of VEGF channels and the binding of surface substances of lymphocytes. The increased permeability of lymphatic vessels is convenient for cancer cells to invade lymphatic vessels and achieve lymphatic diffusion [17–19]. Therefore, VEGF can promote lymphangiogenesis and promote lymphatic metastasis of tumors.

Currently, vascular endothelial cell markers commonly used in tumor angiogenesis studies include CD144, CD31, CD105, CD146 and vWF, etc. [20, 21]. VEGF can stimulate and promote rapid endocytosis of VE cadherin (CD144), a key adhesion molecule of endothelial cells, thereby damaging endothelial barrier function [22]. Vascular endothelial cadherin (VE cadherin) has been reported to play a role in vascular permeability and remodeling. In addition, deletion of VE cadherin or inhibition of its adhesion function can lead to increased vascular permeability [23]. The expression of CD31 is mainly used to prove the existence of endothelial cells, evaluate vascular density, and suggest angiogenesis, which is involved in the occurrence and development of adenoma. VEGF and CD31 expression are positively correlated, and can be used to evaluate tumor angiogenesis together [24]. Based on our in vitro and in vivo tumor tissue experiments, BPTF knockdown not only down-regulated VEGF expression, but also down-regulated the levels of CD31 and VE cadherin in non-small cell lung cancer, suggesting that BPTF can promote the formation of blood vessels in tumor tissues. In human tumor tissues after lung adenocarcinoma surgery, immunofluorescence method and Western Blot detection showed that BPTF, VEGF, CD31 and CD144 were generally overexpressed in cancer tissues compared with adjacent tissues, which further confirmed the possibility of promoting tumor angiogenesis by BPTF-targeted VEGF. We further expanded the sample size and analyzed 75 postoperative cases of lung adenocarcinoma by immunohistochemical staining method. The results showed that the expression of BPTF and VEGF had a significant positive correlation with the correlation coefficient of 0.29, and patients with high expression of BPTF and VEGF had a poor prognosis. Meanwhile, it was found that the overexpression of BPTF and VEGF was correlated with lymph node metastasis and clinical stage. We again proved the correlation between BPTF and VEGF expression and its significance in predicting the prognosis of patients in tumor tissue samples.

In order to further study the clinical significance of BPTF-targeted regulation of VEGF in non-small cell lung cancer, we collected 26 patients who received bevacizumab treatment from January 2018 to January 2021. Immunohistochemical detection showed that BPTF and VEGF were relatively highly expressed in tumor tissues of patients with bevacizumab treatment sensitivity group. The expression of BPTF was relatively low in the tumor tissues of the non-sensitive group treated with bevacizumab, and there was statistical significance between the expression of BPTF and the sensitivity of bevacizumab treatments, that is, patients with the high expression of BPTF embrace good efficacy for bevacizumab treatment, while patients with the low expression of BPTF show poor efficacy of bevacizumab treatment, strongly suggesting that BPTF has great potential as a biomarker for evaluating the efficacy of patients receiving bevacizumab treatment. It must be pointed out that, due to the limited sample size, there was no statistical significance between VEGF expression and bevacizumab efficacy in these 26 patients alone. However, we cannot deny that the proportion of patients with sensitive response still had high VEGF expression. This is basically consistent with the current clinical practice, that is, although bevacizumab is a definite inhibitor of VEGF, VEGF level does not need to be detected before applying bevacizumab. In addition, there have been clinical cases with low expression of VEGF but good efficacy of bevacizumab, and the corresponding responsive mechanisms for this remains to be further studied. Based on the current experimental results, we believe that among patients with lung adenocarcinoma, patients with high expression of BPTF have better efficacy in receiving the VEGF inhibitor bevacizumab therapy, thus providing certain experimental basis for guiding the clinical use of bevacizumab.

In conclusion, from in vitro experiments to in vivo experiments, from cell level to animal level and then to human tissue sample level, this study proved the positive regulation of BPTF on VEGF in NSCLC, and the high expression of BPTF may predict the better efficacy of bevacizumab treatment. Therefore, it provides an important theoretical and experimental basis for developing BPTF/VEGF signaling axis as a candidate therapeutic target and potential prognostic marker of NSCLC.

## Conclusion

In non-small cell lung cancer, BPTF positive regulation of VEGF, BPTF high expression can predict better efficacy of bevacizumab, while BPTF low expression can predict poor efficacy of bevacizumab.

## Data Availability

The datasets generated during and/or analyzed during the current study are available from the corresponding author on reasonable request.
